# EAAT3 promotes amino acid transport and proliferation of porcine intestinal epithelial cells

**DOI:** 10.18632/oncotarget.9583

**Published:** 2016-05-25

**Authors:** Jin-ling Ye, Chun-qi Gao, Xiang-guang Li, Cheng-long Jin, Dan Wang, Gang Shu, Wen-ce Wang, Xiang-feng Kong, Kang Yao, Hui-chao Yan, Xiu-qi Wang

**Affiliations:** ^1^ College of Animal Science, South China Agricultural University/National Engineering Research Center for Breeding Swine Industry, Guangzhou, Guangdong Province, China; ^2^ Institute of Subtropical Agriculture, Chinese Academy of Sciences, Changsha, Hunan Province, China

**Keywords:** excitatory amino acid transporter 3, mammalian target of rapamycin, intestinal epithelial cells, amino acid, proliferation

## Abstract

Excitatory amino acid transporter 3 (EAAT3, encoded by *SLC1A1*) is an epithelial type high-affinity anionic amino acid transporter, and glutamate is the major oxidative fuel for intestinal epithelial cells. This study investigated the effects of EAAT3 on amino acid transport and cell proliferation through activation of the mammalian target of the rapamycin (mTOR) pathway in porcine jejunal epithelial cells (IPEC-J2). Anionic amino acid and cystine (Cys) transport were increased (*P*<0.05) by EAAT3 overexpression and decreased (*P*<0.05) by EAAT3 knockdown rather than other amino acids. MTT and cell counting assays suggested that IPEC-J2 cell proliferation increased (*P*<0.05) with EAAT3 overexpression. Phosphorylation of mTOR (Ser2448), ribosomal protein S6 kinase-1 (S6K1, Thr389) and eukaryotic initiation factor 4E-binding protein-1 (4EBP1, Thr70) was increased by EAAT3 overexpression and decreased by EAAT3 knockdown (*P*<0.05), as were levels of activating transcription factor 4 (ATF4) and cystine/glutamate antiporter (xCT) (*P*<0.05). Our results demonstrate for the first time that EAAT3 facilitates anionic amino acid transport and activates the mTOR pathway, promoting Cys transport and IPEC-J2 cell proliferation.

## INTRODUCTION

Glutamate (Glu) has many important functions in nutrition, metabolism and signaling as a non-essential amino acid [1]. Almost all dietary Glu is extensively metabolized in first-pass by the intestine to maintain intestinal health [2, 3]. Therefore, there is a growing interest in Glu nutrition in mammals [4]. Studies with pigs, rats and mice demonstrate that the excitatory amino acid transporter 3 (EAAT3), encoded by solute carrier family 1 member 1 (*SLC1A1*), is the predominant anionic amino acid transporter in the intestine [5]. In our previous studies, EAAT3 inhibition decreased pig intestinal epithelial cell (IPEC-1) proliferation [6] and intestinal growth in chick embryos [7]. Landeghem, *et al*. [8] reported that EAAT3 expression in microglia cells increased after traumatic brain injury. Collectively, defective EAAT3 expression causes human dicarboxylic aminoaciduria [9] and exacerbates neuronal injury after transient cerebral ischemia [10, 11]. However, the mechanism of EAAT3 action in the intestine is still unclear.

It is widely accepted that amino acid transporters act as carriers and sensors that interact with intracellular nutrient-signaling pathways to regulate metabolism [12]. In particular, the mammalian target of rapamycin (mTOR) pathway is a central regulator that integrates signals from nutrients, especially amino acids, to promote cell proliferation [12, 13]. Interestingly, mTOR increases EAAT3 protein levels in Xenopus oocyte cell membranes and accelerates Glu transport [14].

In the current study we hypothesized that the mTOR pathway is involved in amino acid transport and porcine jejunal epithelial cell (IPEC-J2) proliferation, and that EAAT3 might activate this pathway.

## RESULTS

### Construction of the recombinant plasmid, EAAT3-pcDNA3.1+

**Figure 1:**
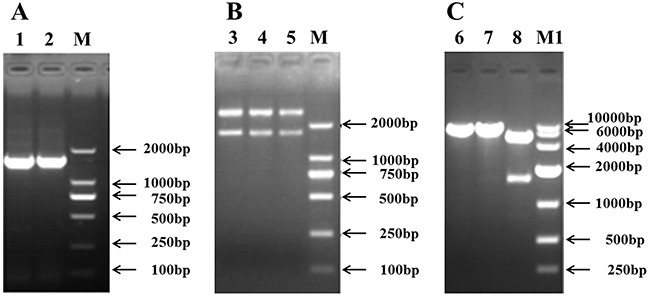
Construction of the recombinant plasmid, EAAT3-pcDNA3.1+ Cloning of the pig EAAT3 cDNA **A.** Identification of the recombinant plasmids EAAT3-pGEM-T **B.** and EAAT3-pcDNA3.1+ **C.** M: DNA Marker 2000; M1: DNA Marker 10000; Lanes 1, 2: PCR products of jejunal excitatory amino acid transporter 3 (EAAT3); Lanes 3, 4, 5: Double enzyme digestions of EAAT3-pGEM-T by *EcoR*I and *Xho*I; Lanes 6, 7: Single enzyme digestions of EAAT3-pcDNA3.1+ by *EcoR*I and *Xho*I, respectively; Lane 8: Double enzyme digestions of EAAT3-pcDNA3.1+ by *EcoR*I and *Xho*I.

The open reading frame (ORF) of the EAAT3 cDNA in Landrace piglets is 1,575 bp in length, encoding a 524-AA polypeptide (Figure 1A). The cDNA sequence shares 99.8% and 99.0% identity with Tibet pig and Huanjiang mini-pig and shares 89.5% and 92.5% with mouse and human, respectively. *EcoR*I and *Xho*I were used for the enzyme digestion identification (Figure 1B–1C); sequencing was also conducted. All analyses confirmed that the pig EAAT3 was successfully inserted into the pcDNA3.1+ vector, creating EAAT3-pcDNA3.1+.

### EAAT3 overexpression and knockdown in IPEC-J2 cells

**Figure 2:**
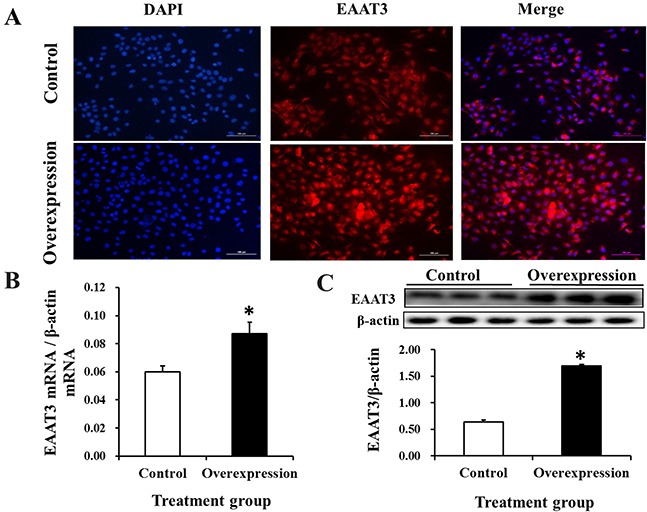
EAAT3 overexpression in IPEC-J2 cells Representative immunofluorescence images of Control and Overexpression cells at 48 h after seeding, labeled with DAPI (blue) and EAAT3 antibody (red) **A.** Scale bars: 100 μm. Control: control group; Overexpression: EAAT3 overexpression group. EAAT3 mRNA abundance (n=6) **B.** and protein level (n=3) **C.** Representative results of three independent experiments are shown as means ± SEM; *P<0.05.

Immunofluorescence microscopy, real-time PCR assays and western blot analyses were performed to assess EAAT3 overexpression in IPEC-J2 cells. Compared with the Control group, EAAT3 fluorescent signaling (Figure 2A) and mRNA (Figure 2B) and protein (Figure 2C) levels increased (*P*<0.05) in the Overexpression group, showing that the Overexpression cells were stably transfected with EAAT3-pcDNA3.1+.

**Figure 3:**
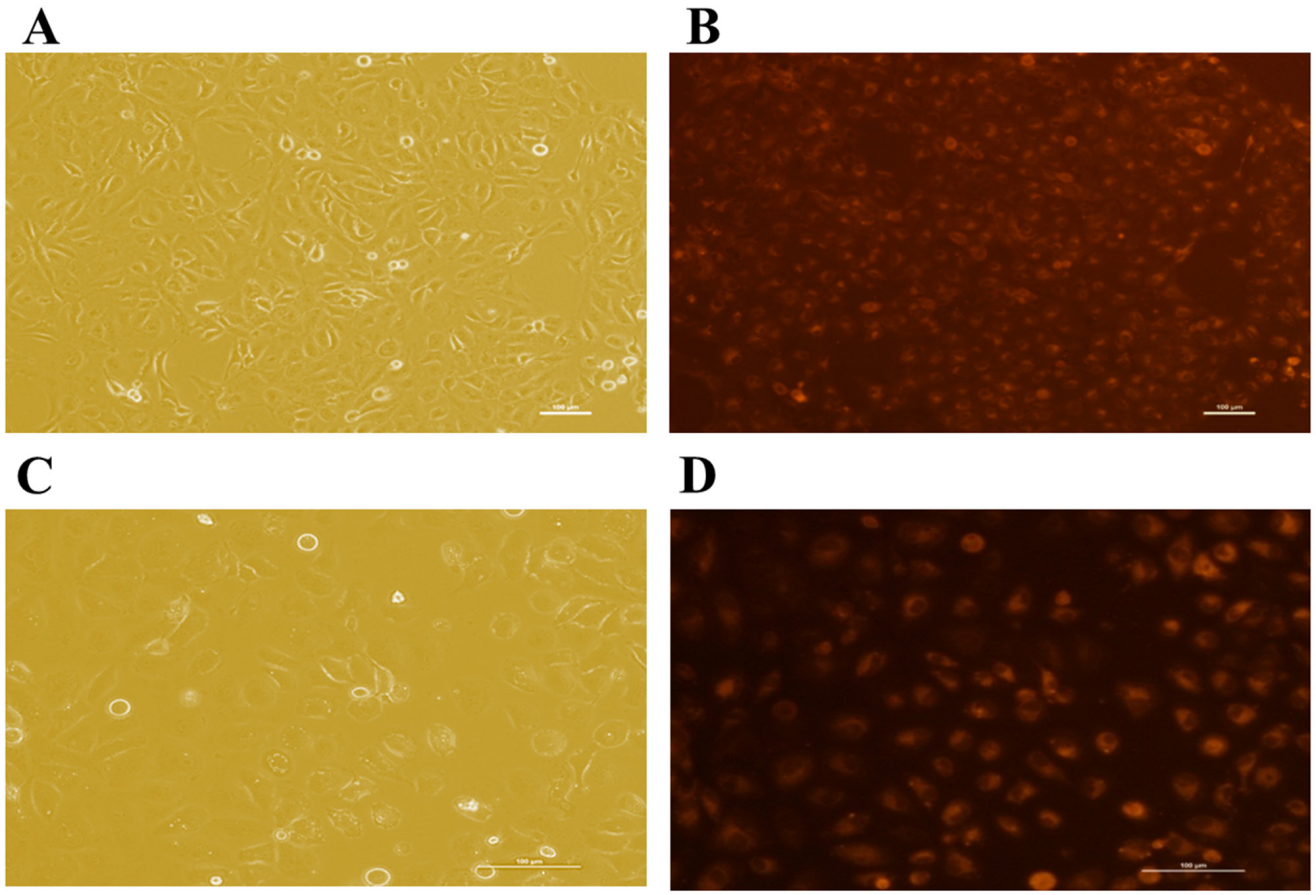
siRNA Representative fluorescence images of IPEC-J2 cells 48 h post-transfection with the transfection control (Cy3)-siRNA IPEC-J2 cells under normal light after transfection with the control (Cy3)-siRNA (100× **A.** and 200× **C.**). Corresponding fluorescence photos of IPEC-J2 cells after transfection with the control (Cy3)-siRNA (100× **B.** and 200× **D.**). Scale bars: 100 μm. Representative results of the three independent experiments are shown.

**Figure 4:**
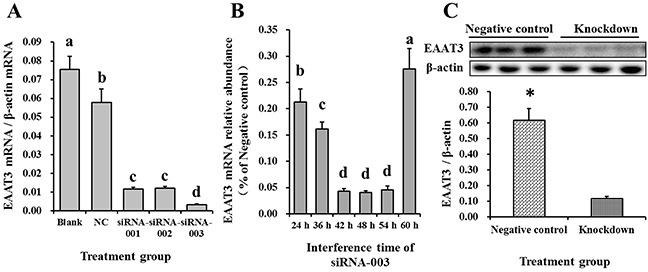
Screening for the optimal EAAT3 siRNA and interference time in IPEC-J2 cells The effects of three siRNAs on EAAT3 mRNA abundance were measured by real-time PCR 48 h post-transfection **A.** Blank: blank control group; NC: negative control group; siRNA-001: EAAT3-siRNA-001 group; siRNA-002: EAAT3-siRNA-002 group; siRNA-003: EAAT3-siRNA-003 group. The effects of siRNA-003 transfection time on EAAT3 mRNA abundance were measured by real-time PCR **B.** Data are expressed as means ± SEM (n=6). Different letters indicate significant differences (*P*<0.05). siRNA-003 treatment reduced EAAT3 protein levels as compared to the Negative controls **C.** Negative control: negative control group; Knockdown: EAAT3-siRNA-003 group. Representative results of three independent experiments are shown. Data are expressed as means ± SEM (n=3); *P<0.05.

To assess siRNA-mediated EAAT3 knockdown in IPEC-J2 cells, we measured transfection levels at different time points via Cy3-labeled siRNA (Cy3-siRNA, RiboBio, Guangzhou, China) (data not shown), a red-fluorescing siRNA. The greatest effect occurred at 48 h post-transfection (Figure 3). Therefore, by measuring EAAT3 expression 48 h post-transfection with the three EAAT3-siRNAs, we found that siRNA-003 produced an optimal interference effect (*P*<0.05; Figure 4A). We also found EAAT3 mRNA abundance in IPEC-J2 cells to be lowest 48 h post-transfection with siRNA-003 (Figure 4B). Compared with Negative control, western blot analysis also showed reduced (*P*<0.05) EAAT3 expression (Figure 4C) in the Knockdown group.

### EAAT3 effects on amino acid transport

**Figure 5:**
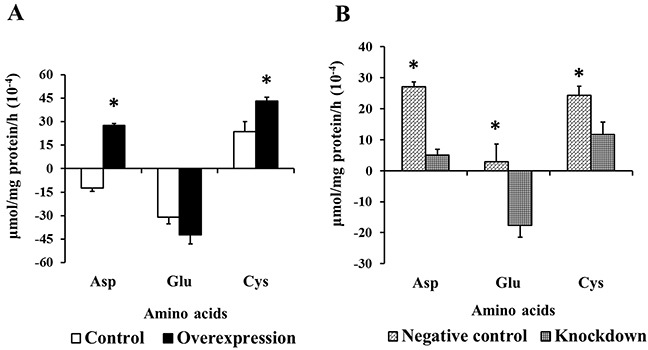
Effects of EAAT3 overexpression A. and knockdown B. on amino acid transport in IPEC-J2 cells Control: control group; Overexpression: EAAT3 overexpression group. Negative control: negative control group; Knockdown: EAAT3-siRNA-003 group. Glu: glutamate; Asp: aspartate; Cys: cystine. Representative results of three independent experiments are shown. Data are expressed as means ± SEM (n=6); *P<0.05.

Free amino acid concentration was measured in IPEC-J2 cells cultured in Advanced DMEM/F-12 (Figure 5). Anionic amino acid and cystine (Cys) transport increased in the Overexpression group compared with Controls (*P*<0.05, Figure 5A), and this effect was significantly reduced by EAAT3 knockdown (*P*<0.05, Figure 5B). EAAT3 overexpression or knockdown had no significant effect on other amino acids.

### EAAT3 overexpression increased IPEC-J2 cell proliferation

**Figure 6:**
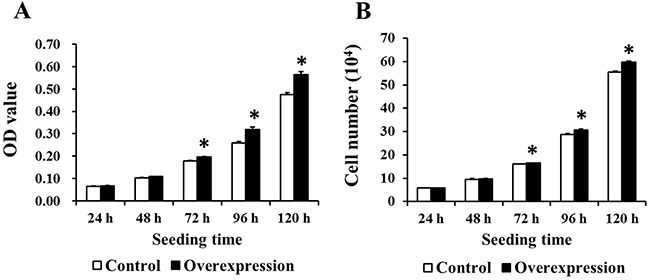
EAAT3 overexpression increased IPEC-J2 cell proliferation The Overexpression group OD values and cell numbers were higher than those of the Control group at 72, 96, and 120 h after seeding, as assessed by MTT (n=20) **A.** and cell counting assay (n=6) **B.** respectively. Control: control group; Overexpression: EAAT3 overexpression group. Representative results of three independent experiments are shown. Data are expressed as means ± SEM; *P<0.05.

MTT and cell count assays were used to evaluate the effects of EAAT3 overexpression on IPEC-J2 cell proliferation. OD values (Figure 6A) and cell numbers (Figure 6B) were increased (*P*<0.05) in the Overexpression group compared to the Control group.

### EAAT3 effects on the mTOR pathway

**Figure 7:**
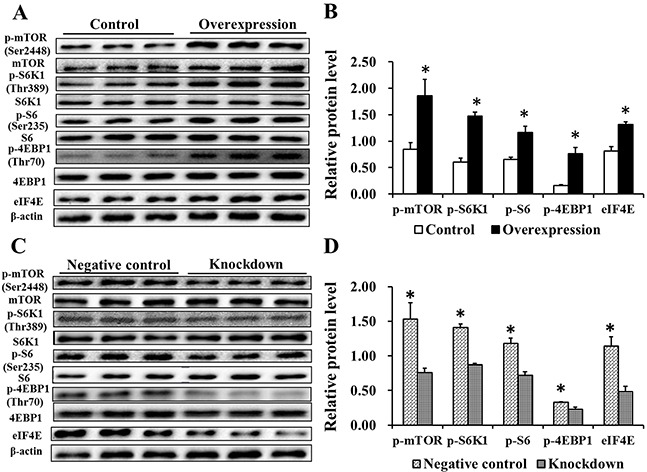
Effects of EAAT3 overexpression A–B. and knockdown C–D. on mTOR pathway related proteins in IPEC-J2 cells Control: control group; Overexpression: EAAT3 overexpression group. Negative control: negative control group; Knockdown: EAAT3-siRNA-003 group. Representative results of three independent experiments are shown. Data are expressed as means ± SEM (n=3); *P<0.05.

We measured the levels of mTOR pathway-related proteins by western blotting. Compared with the Control group, levels of p-mTOR (Ser2448) and its downstream targets, p-S6K1 (Thr389)/p-S6 (Ser235) and p-4EBP1 (Thr70)/eIF4E, were increased in the Overexpression group (*P*<0.05, Figure 7A–7B). Compared with the Negative control group, phosphorylation of mTOR (Ser2448) and its downstream target proteins was decreased (*P*<0.05) in the Knockdown group (Figure 7C–D). These data indicate that EAAT3 could activate the mTOR pathway in IPEC-J2 cells.

### EAAT3 effects on ATF4/xCT expression

**Figure 8:**
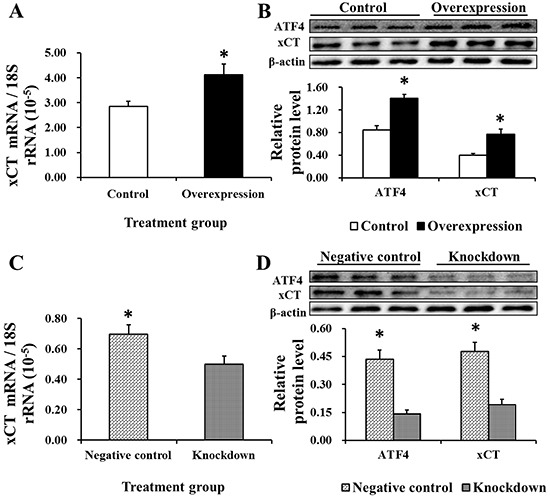
xCT mRNA abundance (n=6) and protein level (n=3) and ATF4 protein level (n=3) after EAAT3 overexpression A–B. or knockdown C–D. as measured by real-time PCR and western blotting Control: control group; Overexpression: EAAT3 overexpression group. Negative control: negative control group; Knockdown: EAAT3-siRNA-003 group. ATF4: activating transcription factor 4; xCT: cystine/glutamate antiporter. Representative results of three independent experiments are shown. Data are expressed as means ± SEM; *P<0.05.

ATF4 and xCT protein levels were measured to help elucidate the underlying mechanism of EAAT3-mediated Cys transport. xCT expression was increased at both the mRNA and protein levels (*P*<0.05) in the Overexpression group as compared with Controls (Figure 8A–8B), and was significantly different between the Negative control and Knockdown groups (Figure 8C–8D). ATF4 protein levels followed the same patterns as those of xCT (Figure 8B & 8D). These data indicate that EAAT3 promotes ATF4 expression and that EAAT3 and xCT levels positively correlate.

## DISCUSSION

**Figure 9:**
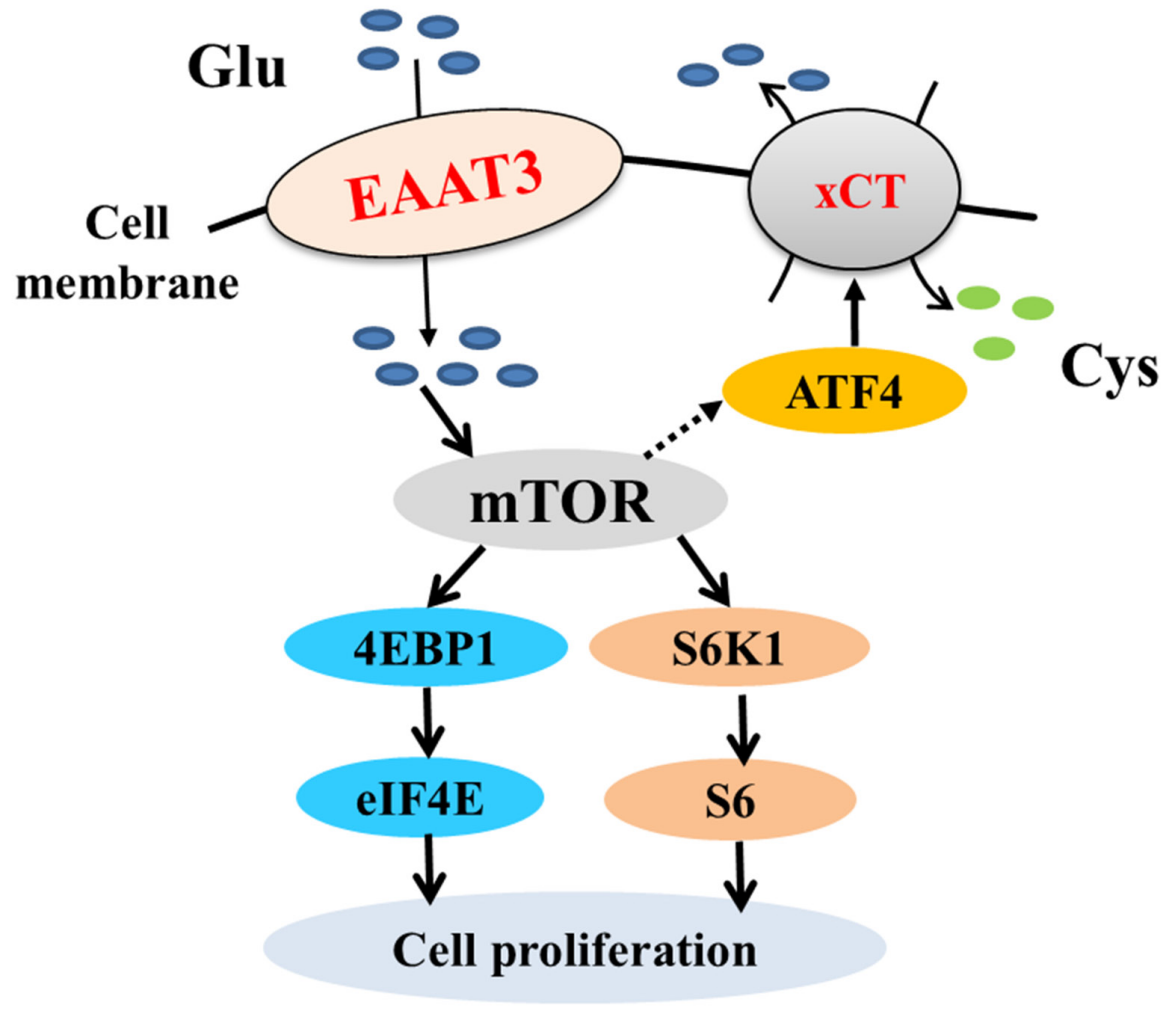
mTOR pathway activation by EAAT3 EAAT3 facilitates Glu transport and activates the mTOR pathway. EAAT3 triggers mTOR pathway activation, which promotes ATF4-mediated xCT expression and might accelerate Cys transport. EAAT3: excitatory amino acid transporter 3; Glu: glutamate; Cys: cystine; mTOR: mammalian target of rapamycin; ATF4: activating transcription factor 4; xCT: cystine/glutamate antiporter.

Glu plays a critical role in metabolism, which regulates a number of physiological processes associated with disease [1, 15]. Glu absorption is mainly mediated by EAAT3 in the nervous system [16], gastrointestinal tract [5, 17] and skeletal muscle [18]. Our previous studies revealed that EAAT3 is a marker of intestinal growth during embryonic development in chicks [7, 19] and pigeons [20–22]. In addition, Glu deficiency or absorptive inhibition of EAAT3 decreased IPEC-1 cell proliferation [6]. A pig intestinal epithelial cell line, IPEC-J2 [23], was used in the present study. Result showed that transport of anionic amino acids and Cys was increased by EAAT3 overexpression and decreased by EAAT3 knockdown while other amino acids were unaffected. Interestingly, proliferation of IPEC-J2 cells in the Overexpression group was increased compared to Controls as determined by MTT and cell counting assays. These results collectively suggested that Glu, aspartate (Asp) and Cys, induced by EAAT3, act as signal transducers to promote cell proliferation (Figure 9).

mTOR is best known for its function in control of cell proliferation, and is typically activated by energy, amino acids and growth factors [24–26]. Notably, amino acid transporters might contribute to initiation of the amino acid-dependent mTOR pathway [27]. Signaling pathways activated by intracellular amino acid concentrations are intrinsically linked to amino acid transporter activity as well as to intracellular amino acid metabolism [28]. We found that phosphorylation of mTOR (Ser2448) and its downstream targets, S6K1 (Thr389) and 4EBP1 (Thr70), was significantly increased and decreased by EAAT3 overexpression and knockdown, respectively. These results demonstrated that EAAT3 contributes to initiation of amino acid-dependent cell signaling in the mTOR pathway (Figure 9).

EAAT3 may be effective in the treatment of advanced renal cell carcinoma by regulating the mTOR pathway as effectively as temsirolimus and everolimus, which are kinase inhibitors of mTOR complex 1 (mTORC1) [29]. mTOR pathway activation may lead to accumulation of hypoxia inducible factor, which promotes the transcription of angiogenesis- and tumor progression-related genes [29]. In addition, EAAT3 has gained attention as a novel therapeutic target due to its association with hypoxia, ischemia and multiple sclerosis [16, 30]. However, whether the effects of EAAT3 in human disease result from its activation of the mTOR pathway requires further study.

The current study showed that Cys transport and expression of both the activating transcription factor 4 (ATF4) and cystine/glutamate antiporter (xCT) were increased following EAAT3 overexpression. Following its activation by free amino acids, mTORC1 might enhance ATF4 expression and subsequently promote expression of amino acid transporters [31]. Furthermore, ATF4 can activate xCT [32, 33]. Indeed, xCT is an obligate exchanger of Cys and Glu, and intracellular Glu depletion inhibits Cys uptake [34, 35]. After uptake by xCT, Cys is rapidly transformed into cysteine, which functions in different pathological conditions by restraining oxidative stress or the inflammatory response [36]. Therefore, we believe that EAAT3 triggers mTOR pathway activation, which promotes ATF4-mediated xCT expression and might accelerate Cys transport (Figure 9).

In summary, our data demonstrate that EAAT3 activates the mTOR pathway to promote amino acid transport and cell proliferation. Moreover, ATF4 and xCT expression may be enhanced during EAAT3-induced mTOR pathway activation to transport excess Glu into the extracellular environment.

## MATERIALS AND METHODS

### Intestinal tissue sample collection

Three male and 3 female 24-d-old Landrace piglets were euthanized with 3% sodium pentobarbital in the dosage of 1 mL/kg before sampling. The entire small intestine was rapidly removed, washed, and frozen in liquid nitrogen. All procedures were approved by the Animal Care Committee of the South China Agricultural University (Guangzhou, China).

### Cloning and plasmid construction

Total RNA was isolated from the jejunum using the TRIzol reagent (Invitrogen, Carlsbad, CA) following the manufacturer's instructions. RNA quality and quantity were determined by ultraviolet spectroscopy using a NanoDrop ^®^ND-2000 (Thermo Fisher Scientific, DE, USA). Pig full-length EAAT3 cDNA was amplified by reverse transcription polymerase chain reaction (RT-PCR) using mRNA extracted from the jejunum. Designed primer sequences are shown in Table 1. The EAAT3 DNA fragment was cloned into the *EcoR*I and *Xho*I sites of the pcDNA3.1+ vector to generate the EAAT3-pcDNA3.1+ recombinant plasmid. The ligation product was transferred into DH5α *E. coli* cells and spread on an anti-ampicillin Luria-Bertani medium plate cultured at 37°C for 12–16 h. Several clones were then chosen from the plate and plasmids were extracted for PCR, enzyme digestion and sequencing. Purification was performed using the Endo-Free Plasmid Kit (TIANGEN, Beijing, China) according to the manufacturer's instructions.

**Table 1:** Primers used for cloning and quantitative real-time PCR

Genes (accession no)	Primers	Sequences (5′–3′)	Size (bp)
EAAT3 (1)	sense	ATGGGGAAACCGGCGAGGA	1575
	antisense	CTAGAACTGGGAGGTCTGGGTGAATGA	
EAAT3 (2)	sense	GGCACCGCACTCTACGAAGCA	177
	antisense	GCCCACGGCACTTAGCACGA	
xCT (KJ893432.1)	sense	GGTCAGAAAGCCTGTTGT	190
	antisense	GATGAAGATTCCTGCTCC	
18S rRNA (NR_046261.1)	sense	AATTCCGATAACGAACGAGACT	145
	antisense	GGACATCTAAGGGCATCACAG	
β-actin (XM_003357928)	sense	TGCGGGACATCAAGGAGAAG	216
	antisense	AGTTGAAGGTGGTCTCGTGG	

EAAT3: excitatory amino acid transporter 3, EAAT3 (1) was used for cloning and EAAT3 (2) for real-time PCR analysis, 1575 the size was obtained from sequencing.

### Design and synthesis of EAAT3 small interfering RNA

Three siRNAs targeting *SLC1A1* and an unrelated negative control siRNA were purified via high performance liquid chromatography (RiboBio, Guangzhou, China) (Table 2). Twenty-four hours prior to transfection, IPEC-J2 cells were plated into a 6-well plate at 30%–50% confluence. siRNAs (50 nmol) were transfected into IPEC-J2 cells according to the manufacturer's instructions.

**Table 2:** Sequences of chemically synthesized EAAT3-siRNAs

Name	Type	Sequence
siRNA-001-sense	RNA	5′GCAAUCCACUCCAUUGUUA dTdT 3′
siRNA-001-antisense	RNA	3′ dTdT CGUUAGGUGAGGUAACAAU 5′
siRNA-002-sense	RNA	5′ GCAGUGGCAGCUGUGUUUA dTdT 3′
siRNA-002-antisense	RNA	3′dTdT CGUCACCGUCGACACAAAU 5′
siRNA-003-sense	RNA	5′ CCAAGAAGUCCUAUGUCAA dTdT 3′
siRNA-003-antisense	RNA	3′ dTdT GGUUCUUCAGGAUACAGUU 5′

EAAT3: excitatory amino acid transporter 3.

### Cell culture and transfection

IPEC-J2 cells, a porcine intestinal epithelial cell line derived from the jejunal crypts of neonatal piglet, was kindly provided by Dr. Yu-long Yin (University of Chinese Academy of Sciences). Cells were cultured in 6-well plates (#3516, Corning, NY, USA) in DMEM/F12 medium (Thermo, Waltham, MA, Cat: 12400-024) supplemented with 5% fetal bovine serum (FBS, Gibco, Carlsbad, CA, Cat: 10099-141), 1% penicillin/streptomycin, 5 μg/L Insulin-Transferrin-Selenium (Sciencell, Carlsbad, CA, USA, Cat: 0803) and 5 μg/L epidermal growth factor (Sciencell, Carlsbad, CA, USA, Cat: 105-04) at 37°C in 5% CO_2_. Medium was changed every two days. All experiments were performed in triplicate on cell passages 10–20.

IPEC-J2 cells were seeded at 1×10^5^ cells/mL into 6-well cell culture plates and transfected with the pcDNA3.1+ or EAAT3-pcDNA3.1+ plasmid and siRNAs using Lipofectamine™ 3000 (Invitrogen Life Technologies, Carlsbad, CA, USA) on the following day, according to the manufacturer's recommendations. G418 (800 μg/mL, tested previously before experiment) was added to the medium after transfection. After seven days, G418 levels decreased to 400 μg/mL and nontransfected cells all died. Fourteen days later, surviving clones were analyzed, and the positive clones were collected. In the following experiments, cells transfected with pcDNA3.1+ are the Control group and cells transfected with EAAT3-pcDNA3.1+ are the EAAT3 Overexpression group. Cells transfected with the unrelated control siRNA are the Negative control group and cells transfected with EAAT3-siRNA are the Knockdown group.

### Immunofluorescence microscopy

pcDNA3.1+ and EAAT3-pcDNA3.1+ stably transfected IPEC-J2 cells were seeded at 1×10^4^ cells/mL into 96-well culture plates and fixed with 4% paraformaldehyde for 30 min. Cells were then permeabilized with 0.3% Triton X-100 for 15 min and blocked in a protein solution (Dako, Carpinteria, CA) for 20 min. The primary antibody, anti-EAAT3 (1:500 in antibody diluent; Dako, Carpinteria, CA), was applied to these cells for 2 h at room temperature. Secondary staining was performed with Cy3 conjugated antibodies (Jackson ImmunoResearch, West Grove, PA) (1:200 in antibody diluent) with incubation at room temperature for 1 h. Nuclei were stained with 4, 6 diamidino-2-phenylindole (DAPI, 1:1000 in PBS; Sigma, St. Louis, USA), for 5 min at room temperature. Fluorescence signals were observed with a fluorescence microscope (NIS-Elements, Nikon, Japan). At least three independent experiments were performed to verify results.

### Real-time polymerase chain reaction (real-time PCR)

mRNA abundance (n=6) was determined by real-time PCR using a Stratagene MxPro 3005P apparatus (Agilent Technologies, Santa Clara, CA) and the SYBR Green Real-Time PCR Master Mix (TOYOBO, Tokyo, Japan). Specific primer pairs were designed using Primer 5.0 software (Table 1). A melting curve analysis was conducted to confirm the specificity of each product, and product sizes were verified in ethidium bromide-stained 1.5% agarose gels in Tris acetate-EDTA buffer. Quantitative data were collected using the 2^−ΔΔCt^ method. The experiment was performed in triplicate.

### Experimental treatment and determination of the free amino acids

After reaching 80%–90% confluence in DMEM/F12 containing 5% FBS, cells were starved for 2 h in an amino acid-deprived medium with Earle's Balanced Salt Solution (EBSS) (Sigma, St. louis, MO, USA, Cat: E2888) and a vitamin mixture (Sigma, St. louis, MO, USA, Cat: R7256) according to established protocols [37]. Cells were then cultured in a serum- and Advanced DMEM/F-12 (Thermo, Waltham, MA, Cat: 12634-028) for 4 h. Afterwards, cells were analyzed via western blotting and the free amino acid concentration was tested in culture medium using a Hitachi L-8900 amino acid analyzer (Hitachi, Tokyo, Japan).

### MTT assay

Control and Overexpression cells were cultured in 96-well plates (#3599, Corning, NY, USA) at 1×10^4^ cells/mL in growth medium. Cell numbers were quantified at different time points (24, 48, 72, 96, and 120 h) by incubating cells in 5 mg/ml 3-(4,5-dimethylthiazol-2-yl)-2,5-diphenyltetrazolium bromide (MTT, Sigma, St Louis, MO, USA) for 4 h. Crystals were dissolved in dimethylsulfoxide (DMSO). OD was evaluated in an ELISA reader at a wavelength of 490 nm. Results were confirmed in three independent experiments with 20 samples per treatment.

### Cell count assay

Control and Overexpression cells were cultured in 6-well plates at 1×10^5^ cells/mL in growth medium. Briefly, cells were detached with 0.25% trypsin (Sigma, St Louis, MO, USA) for 5–8 min at 37°C after washing twice with PBS and then blocked with an equal volume of growth medium. The number of viable cells was determined using a hemocytometer under an Inversion Microscope System (Nikon, Japan). Three independent experiments were conducted.

### Western blot analysis

IPEC-J2 cells were lysed in RIPA buffer containing 1% Triton-X, 20 mmol/L Tris-HCl (pH=7.4), 5 mmol/L EDTA and 1 mmol/L phenyl-methylsulfonyl fluoride (PMSF) followed by 14,000×*g* centrifugation at 4°C for 15 min. Supernatant protein concentrations were determined using a BCA Protein Assay Reagent Kit (Thermo Fisher Scientific, Rockford, USA). Proteins (10 μg) were separated on 8–10% SDS/PAGE gels and then transferred onto PVDF membranes (Millipore, Billerica, MA). After blocking, membranes were incubated with a primary antibody. Anti-mTOR (#2972), anti-phospho-mTOR (Ser2448, #5536), anti-S6K1 (#9202), anti-phospho-S6K1 (Thr389, #9205), anti-S6 (#2708), anti-phospho-S6 (Ser235/236, #9234), anti-4EBP1 (#9644), and anti-phospho-4EBP1 (Thr70, #2855) antibodies were obtained from Cell Signaling Technology (Beverly, MA, USA); anti-EAAT3 (sc-25658) and anti-ATF4 (sc-200) antibodies were obtained from Santa Cruz (Dallas, Texas, USA); anti-xCT (ab60171) and anti-eIF4E antibodies were obtained from Abcam (Cambridge, MA, USA); anti-β-actin (AP0060), anti-rabbit IgG (BS13278) and anti-goat IgG (BS30503) antibodies were obtained from Bioworld Technology (Louis Park, MN, USA). Proteins were visualized using the Beyo ECL Plus chemiluminescence detection kit (Beyotime Institute of Biotechnology, Beyotime, Shanghai, China). Enhanced chemiluminescence (ECL) signals were scanned using a FluorChem M apparatus (Protein Simple, Inc., Santa Clara, CA). Band density was analyzed using Image Analysis Software (Tanon, Shanghai, China).

### Statistical analysis

Data are from at least three independent experiments and are expressed as the mean ± SEM. Student's t test was conducted to determine the differences between 2 groups using SAS (Version 9.2; SAS Inst. Inc., Cary, NC). Differences among groups were determined using Duncan's multiple-range test. Differences between treatments were considered statistically significant when *P*<0.05.
